# The Balance between Orthodontic Force and Radiation in the Jawbone: Microstructural, Histological, and Molecular Study in a Rat Model

**DOI:** 10.3390/biology10111203

**Published:** 2021-11-18

**Authors:** Hadas Dorchin-Ashkenazi, Ravit Ginat-Koton, Yankel Gabet, Yehuda Klein, Stella Chaushu, Hezi Dorchin, Tamar Brosh, Marilena Vered

**Affiliations:** 1 Department of Orthodontics, School of Dental Medicine, Tel Aviv University, Tel Aviv 69978, Israel; hdorchin@gmail.com; 2 School of Dental Medicine, Tel Aviv University, Tel Aviv 69978, Israel; ginat.ravit@gmail.com; 3 Department of Anatomy and Anthropology, Sackler Faculty of Medicine, Tel Aviv 69978, Israel; yankel@tauex.tau.ac.il; 4 Department of Orthodontics, Hebrew University-Hadassah Faculty of Dental Medicine, Jerusalem 91120, Israel; Yehuda.klein@mail.huji.ac.il (Y.K.); drchaushu@hadassah.org.il (S.C.); 5 The Institute of Dental Sciences, Hebrew University, Hadassah Medical Center, Jerusalem 91120, Israel; 6 Independent Researcher, Haifa 3461717, Israel; dorchin@017.net.il; 7 Department of Oral Biology, School of Dental Medicine, Tel Aviv University, Tel Aviv 69978, Israel; tbrosh@tauex.tau.ac.il; 8 Department of Oral Pathology, Oral Medicine and Maxillofacial Imaging, School of Dental Medicine, Tel Aviv University, Tel Aviv 69978, Israel; 9 Institute of Pathology, Sheba Medical Center, Tel Hashomer, Ramat Gan 52621, Israel

**Keywords:** orthodontic tooth movement, rats, maxilla, radiation, micro-CT, osteoclasts, osteoblasts

## Abstract

**Simple Summary:**

Patients with head and neck cancer are frequently treated by radiation, which results in a lifelong risk of damage (necrosis) to the jawbones. Some of the irradiated young patients at a later time in life may be interested in orthodontic treatment for esthetic or functional purposes. We undertook this study in order to investigate changes that occur in irradiated jawbones when mild orthodontic force is applied in a rat laboratory model. We found that one low dose of radiation had negatively affected the jawbones and that these changes were visible in X-ray images as well as in microscopic slides. The irradiated bones seemed to be denser in the X-rays and had fewer cells that usually regulate normal bone turnover, compared to non-irradiated bones. However, when orthodontic force was applied after radiation, the changes in the irradiated bones were largely, but not completely, reversed in both X-rays and microscopy to the point that bone properties were approaching those of non-irradiated, orthodontically treated, bones. The findings of this study indicate that orthodontic force may have a beneficial effect on the maintenance of jawbone vitality after radiation, but additional studies using different time-lags between radiation and orthodontic force and higher radiation doses are warranted to support these findings.

**Abstract:**

Irradiation of facial bones is associated with a lifelong risk of osteonecrosis. In a rat model, maxillae were exposed to a single 5 Gy dose of external beam radiation and orthodontic force was applied for 2 weeks on the first maxillary molar; control rats were treated identically without radiation. Tooth movement in irradiated jaws was 30% less than in controls, representing radiation-related damage. Micro-CT, histological, and molecular outcomes of orthodontic tooth movement were studied. Microstructurally, bone parameters (trabecular thickness, bone volume fraction, bone mineral density) were significantly affected by orthodontic force but not by radiation. Histological parameters were influenced only by orthodontic force, especially by an increase in osteoclasts. A molecular study revealed a differential distribution of cells expressing pre-osteoclast markers (RANK+—majority, CD11b+, CD14+—minority), with changes being influenced by orthodontic force (increased CD11b+ and CD14+ cells) and also by radiation (decreased RANK+ cells). The activation status of osteoclasts (TRAP staining) showed an orthodontic-force-related increase, which probably could not fully compensate for the radiation-associated impairment. The overall balance showed that orthodontic force had elicited a substantial microstructural, histological, and functional normalization process in irradiated maxillae but a radiation-induced impact was still conspicuous. Additional studies are needed to validate these findings.

## 1. Introduction

The recently reported improved survival rates of head and neck post-irradiated cancer patients, mainly those with tonsillar and laryngeal cancers [1,2], have resulted in a growing patient population that requires unique clinical considerations in orthodontic treatment. Previous studies on orthodontic treatment in post-irradiated cancer survivors have mainly focused on tooth/root morphological and developmental anomalies [3,4,5]. However, the effects of irradiated bone on orthodontic tooth movement, and vice versa, have not been investigated. Changes in irradiated bone metabolism are closely related to orthodontic treatment because both entail bone remodeling.

Healthy bone is a dynamic tissue, with continual, well-coordinated, coupled resorption and formation of bone [6]. Application of orthodontic force uncouples these processes by inducing early changes at pressure sites of bone resorption and inhibition of bone formation and later changes at tension sites, by inhibiting resorption and stimulating bone formation [7]. Orthodontic tooth movement can also be affected by changes in bone-related factors that are located within the dental pulp of orthodontically treated teeth [8].

The most common morbidity of jawbones located in the field of radiation is osteoradionecrosis, which may be regarded as a unique sequela of direct irradiation damage to the bone (impaired remodeling) combined with the setting of dentition-related factors and oral microbiota [9,10,11]. There is an increased lifelong risk of osteoradionecrosis [3,6]. The pathophysiology is complex, and the theories suggested to explain osteoradionecrosis have been reviewed well [12]. These theories have addressed the impact that radiation has on bone-related cells (osteoblasts, osteocytes, osteoclasts), endothelial cells, fibroblasts, extracellular matrix, and inflammation-related mediators. Although our current knowledge of osteoradionecrosis has been remarkably enriched over the years, it is still not sufficient to allow us to prevent or cure it.

To better understand the inter-relations between orthodontic force and radiation, we employed an animal model to investigate the microstructural and histological changes induced by orthodontic tooth movement in irradiated maxillae and to assess molecular changes in bone-forming and bone-resorbing cells and their progenitors.

## 2. Materials and Methods

### 2.1. Animal Model

The study comprised twenty-one 14-week-old Sprague Dawley male rats (Envigo, Jerusalem, Israel) and was approved by the Committee of the Veterinary Service Center of the Faculty of Medicine, Tel Aviv University (M-13-071). It was performed in conformation with the ARRIVE guidelines for the reporting of animal studies (available online at https://arriveguidelines.org (accessed on 3 April 2014).

After acclimatization (1 week), the animals were housed in open cages in groups of four and maintained on a standard 12 h/12 h light/dark cycle. Tap water, sterilized food, (soaked in water, especially after radiation and/or after introduction of the orthodontic appliance) ad libitum (Rodent Diet 2018c, Harlan-Teklad, Madison, WI, USA), and sterilized laboratory animal bedding (Sani-chips 7090, Harlan-Teklad) were supplied.

### 2.2. Irradiation

Eleven rats were randomly selected for irradiation. A single dose of 5 Gy was applied to the head. Irradiation was given by an external X-ray beam (YXLON.TU 160-D02, End Grounded Metal–Ceramic X-ray Tube, Switzerland), while the rest of the body was protected by a lead apron. The remaining rats (*N* = 10) were handled similarly but without turning on the X-ray beam. A dose of 7 Gy delivered in five daily fractions in rats is bioequivalent to the clinical dose that head and neck cancer patients receive according to standard protocol [13]. Accordingly, the 5 Gy dose given in the present animal study was bioequivalent to 75% of the routine clinical dose.

### 2.3. Application of Orthodontic Force

After allowing the rats a 2-week recovery period, a Sentalloy NiTi closed coil spring (25 cN, Dentsply GAC, Islandia, NY, USA) was installed for 2 weeks in a split-mouth design. The left first maxillary molar was orthodontically moved mesially, while the upper incisors served as an anchoring unit. The right first maxillary molar served as an internal control (i.e., there was no orthodontic tooth movement). Therefore, the study comprised controls (no radiation, *N* = 10) and irradiated (*N* = 11) groups. In each group, orthodontic tooth movement was performed on the left first maxillary molar, while the right first maxillary molar was not subjected to orthodontic force.

All radiation and orthodontic procedures were performed under general anesthesia with ketamine (100 mg/kg, Clorketam, Vetquinol, France) and xylazine (10 mg/kg, Sedaxylan Veterinary, Eurovet Animal Health BV, Bladel, The Netherlands). During the 2 weeks of orthodontic force application, follow-up procedures were performed twice a week under anesthesia (ketamine 50 mg/kg), during which the crowns of the mandibular incisors were reduced to the gingival level in order to avoid dislocation of the orthodontic appliance [14], and the rats were weighed. Within 24 h from the end of the 2-week orthodontic force application, rats were euthanized by CO_2_ and the maxillae were dissected and fixed in a 4% buffered paraformaldehyde solution (Gadot, Netanya, Israel).

### 2.4. Micro-CT Analysis

**Figure 1:**
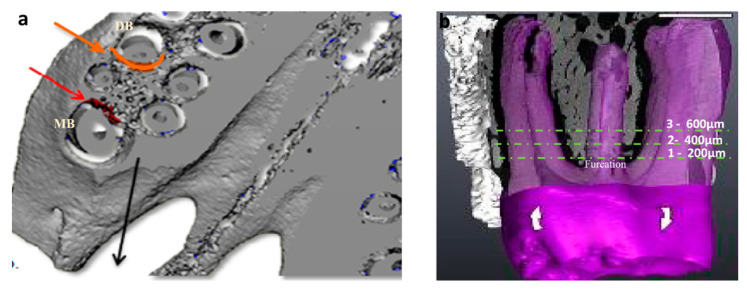
(**a**) Micro-CT section that illustrates the tension and pressure areas around the roots of the maxillary left first molar. The orange arrow and line show the pressure area in relation to the disto-buccal (DB) root, and the red arrow and line indicate the tension area in relation to the mesio-buccal (MB) root. The black arrow indicates the direction of the orthodontic tooth movement; (**b**) An illustration of the locations of the histological sections. Sections were performed on a transversal plane of the maxilla, and each section contained the entire maxillary jawbone. The sections were taken at three locations (1, 2, 3) through the roots of the first maxillary molar: location 1 = 200 µm apical to the root furcation, location 2 = 400 µm, and location 3 = 600 µm. This yielded a total of three slides per maxilla.

The harvested maxillae were micro-CT scanned (μCT50, Scanco, Switzerland). Transverse-oriented sections of 17.2 µm width were made along the mesio-buccal and disto-buccal roots (Figure 1a). The mesial aspect of the disto-buccal root was considered as the pressure side, and the distal aspect of the mesio-buccal root was considered as the tension side according to the principals of orthodontic tooth movement and subsequent histological changes, as previously described [7,15,16]. The bony area to be analyzed around those roots started at 344 µm under the root furcation and extended apically for a total of 516 µm, since the formation of tension and pressure areas were assumed to have a center of rotation located at the tooth furcation [17] (Figure 1b). The assessed parameters included the distance of the orthodontic tooth movement (µm) relative to the second upper molar as well as the microstructure-related volumetric bone mineral density (mgHA/cm^3^), the trabecular bone volume fraction out of the total volume (%), and the trabecular thickness (mm). In the left side of the maxillae (to which orthodontic force was applied) of both control and irradiated rats, the microstructural parameters were assessed in two regions of the first molar corresponding to the pressure and tension sides. In the right side of the maxillae (to which no orthodontic force application) of both control and irradiated rats without true pressure or tension sides, the values of the parameters in the areas corresponding to these sides were averaged.

### 2.5. Histological Preparation

After being scanned, the maxillae were decalcified in 10% ethylene diamine-tetra-acetic acid (Titriplex III, Billerica, MA, USA) for 6 weeks, embedded in paraffin, and cut into three micron-thick sections for the preparation of hematoxylin- and eosin-stained slides and histochemically and immunohistochemically stained slides. Sections were performed on a transversal plane of the maxilla, and each section contained the entire maxillary jawbone. These sections were performed at the appropriate levels of the micro distance of each of the three transversal sections relative to the furcation, as illustrated in Figure 1b.

### 2.6. Histomorphometric Analysis

Histomorphometry (hematoxylin- and eosin-stained slides, light microscope, Olympus BH-2, Tokyo, Japan) was performed on a transversal plane of the maxilla, in analogue planes and distances at which the maxillae were micro-CT scanned. Photomicrographs were taken in a systematic manner by placing the upper-right corner of the field on the periphery of the tooth root and with the rest of the field overlying the periodontal ligament and the adjacent alveolar bone. The histomorphometric method was an adaptation of the point-counting procedure [18,19] involving a camera (Olympus DP70, Tokyo, Japan) and then transported to a full-screen PowerPoint (ppt) slide. We used a simple python script to overlap a 10 × 10 square grid on top of each ppt slide. Point counting was performed on bone, connective tissue, blood vessels, and inflammation. Whenever the graticule-square center (marked by a “+”) hit one of the four parameters, that parameter scored 1 point. The sum of points overlying each hit parameter in each case was calculated and expressed as the area fraction percentage of each parameter as a part of the total number of “+” summed in all sections comprising the entire section area. The results were represented as a mean area fraction percentage for each parameter in each study group. In addition, osteoblasts and osteoclasts overlapped by the “+” were counted in each of the sections prepared for each case and expressed as a mean number per field for each of the study groups. An osteoblast was defined morphologically as a round-shaped mononuclear cell that lined bone surfaces, and an osteoclast was defined as a multinucleated cell found in a resorptive bone lacuna on bone surface.

### 2.7. Immunohistochemical Stains

All procedures were performed using double staining in order to identify the precursors of osteoblasts (mesenchymal stem cell origin), as well as the precursors of osteoclasts (hematopoietic stem cell origin). A total of six different markers were used, three for each type of cell. Each slide was double-stained, with one type of antibody aimed at identifying cells of a pre-osteoclast type and the other type of antibody aimed at identifying cells of a pre-osteoblast type. The coupling of these antibodies and their dilutions are detailed in Table 1. This technique requires that the primary antibodies be prepared from different origins and that each of them target a different cell compartment. Heat-induced epitope retrieval buffer (Titriplex III, Billerica, MA, USA; pH = 9) was the first stage of pre-treatment retrieval, followed by 10 min in a pressure cooker.

The secondary antibodies were horseradish peroxidase for the primary anti-rabbit antibodies (ZUCO32-100, Zytomed Systems, Berlin, Germany) and for the anti-mouse primary antibodies (POL2DS-006, Zytomed Systems) and alkaline phosphatase polymer antibody for the anti-rabbit primary antibodies (POL2DS-006, Zytomed Systems). Since the primary antibodies to both pre-osteoclasts and pre-osteoblasts required the same pre-treatment, we used 3,3′-diaminobenzidine (Invitrogen, Waltham, MA, USA) for the first chromogenic step (brown chromogen) and Permanent AP Red (Zytomed Systems) (pink-purple chromogen) for the second primary antibody.

### 2.8. Immunomorphometry

The immunomorphometric analysis was conducted similarly to the histomorphometric study. Photomicrographs were taken at ×200, and photography followed the same systematic methodology as for the histomorphometry.

We used a 16 × 16 square grid on top of each photomicrograph. Whenever the “+” overlapped either a brown- or a pink-purple-stained cell/extracellular component in the periodontal ligament area, it was considered as a positive hit and a value of 1 point was given to that specific parameter. The “+” that overlapped the tooth root and alveolar bone were eliminated from the total 256 “+” per grid, leaving only the effective number of “+” to be further considered. We then calculated the percent of the brown-stained Tenascin, receptor activator of nuclear factor kappa B (RANK) and the secreted protein acidic and rich in cysteine (SPARC) or purple-stained CD14, periostin, and CD11b components that overlapped a “+” from the effective number of “+” per section. The three levels per tooth were averaged, and the results were expressed as the mean number of positively stained cells per pressure or tension sides per right or left first molar in each study group.

### 2.9. Tartrate-Resistant Acid Phosphatase (TRAP) Staining and Assessment

Osteoclast activity was detected by TRAP staining (Sigma-Aldrich #387A; Saint Louis, MO, USA) with hematoxylin counter-staining according to the manufacturer’s instructions as previously described [27] on 5 µ thick formalin-fixed and paraffin-embedded sections. Assessment was done exactly as for the immunohistochemically stained slides.

### 2.10. Statistical Analysis

The effect of radiation and orthodontic force (separately and their interactions) on the microstructural parameters of volumetric bone mineral density, trabecular bone volume fraction, and trabecular thickness in the pressure and tension sides was analyzed with ANOVA with repeated measures. The same test was used to analyze how radiation and orthodontic force affect the area fraction percent of the histomorphometric parameters of bone, connective tissue, blood vessels, inflammation, and the mean numbers of osteoblasts and osteoclasts, as well as the mean numbers of cells expressing immunohistochemical markers of pre-osteoclasts (RANK, CD14, and CD11b) and pre-osteoblasts (SPARC, periostin, tenascin) and the mean number of cells positive for TRAP stain. Analyses were performed using SPSS software, version 27.0 (Chicago, IL, USA). Statistical significance was set at *p* < 0.05.

## 3. Results

### 3.1. General

The rats’ weight in the controls at the beginning and end of the experiments did not change significantly (394.7 ± 52.4 g and 377.7 ± 51.6 g, respectively; *p* > 0.05), similarly to what we found in the irradiated rats (403.3 ± 26.4 g and 390.8 ± 27.1 g, respectively; *p* > 0.05).

### 3.2. Micro-CT Analysis

The distance of orthodontic tooth movement in irradiated rats was 220.4 ± 81.7 µm, which was 30% lower than the distance of 313.0 ± 76.2 µm of the controls (*p* = 0.002).

**Figure 2:**
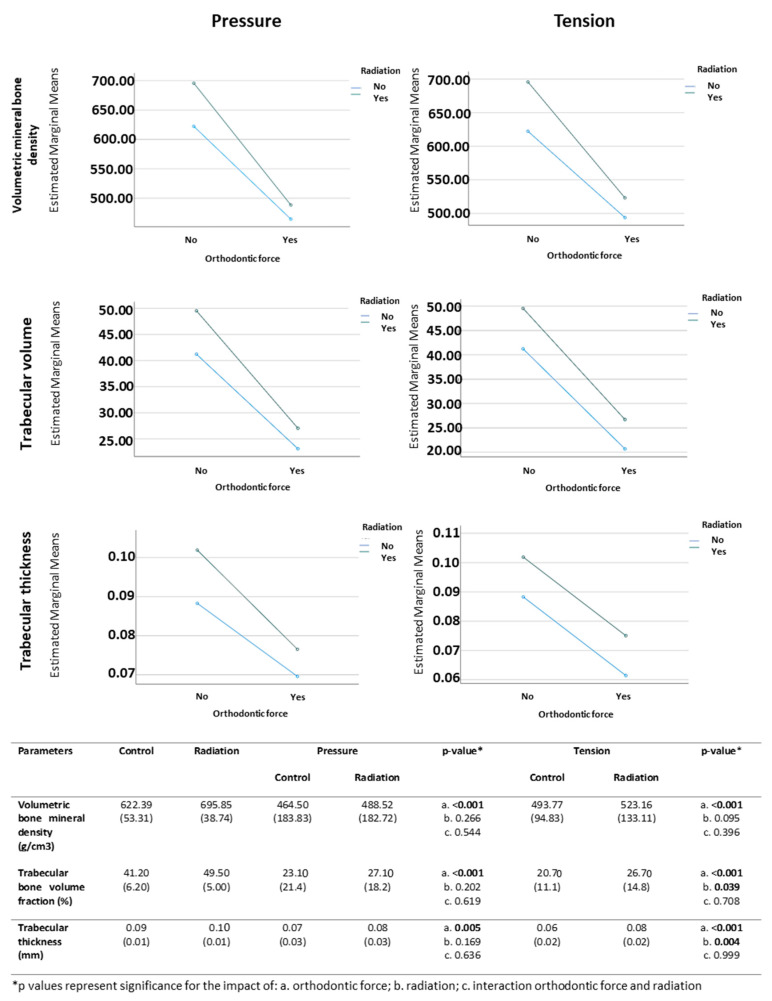
Microstructural changes in volumetric mineral bone density, trabecular volume, and trabecular thickness as a factor of orthodontic force and radiation.

In the pressure side, all evaluated parameters (i.e., volumetric bone mineral density, trabecular bone volume fraction and trabecular thickness) were significantly affected by orthodontic force but not by radiation, with mean values being lower in association with orthodontic tooth movement compared to those associated with no application of orthodontic force (Figure 2). Similarly, in the tension side, all evaluated parameters were significantly affected by the orthodontic force but not by radiation, with the mean values being lower in association with orthodontic tooth movement compared to those when no orthodontic force was applied (Figure 2). There was also a significant effect of radiation on trabecular bone volume fraction and trabecular thickness: mean values were higher in association with radiation compared to those when no radiation was applied (Figure 2). However, no significant interactions were found between the use of orthodontic force and radiation.

### 3.3. Histomorphometric Analysis

**Figure 3:**
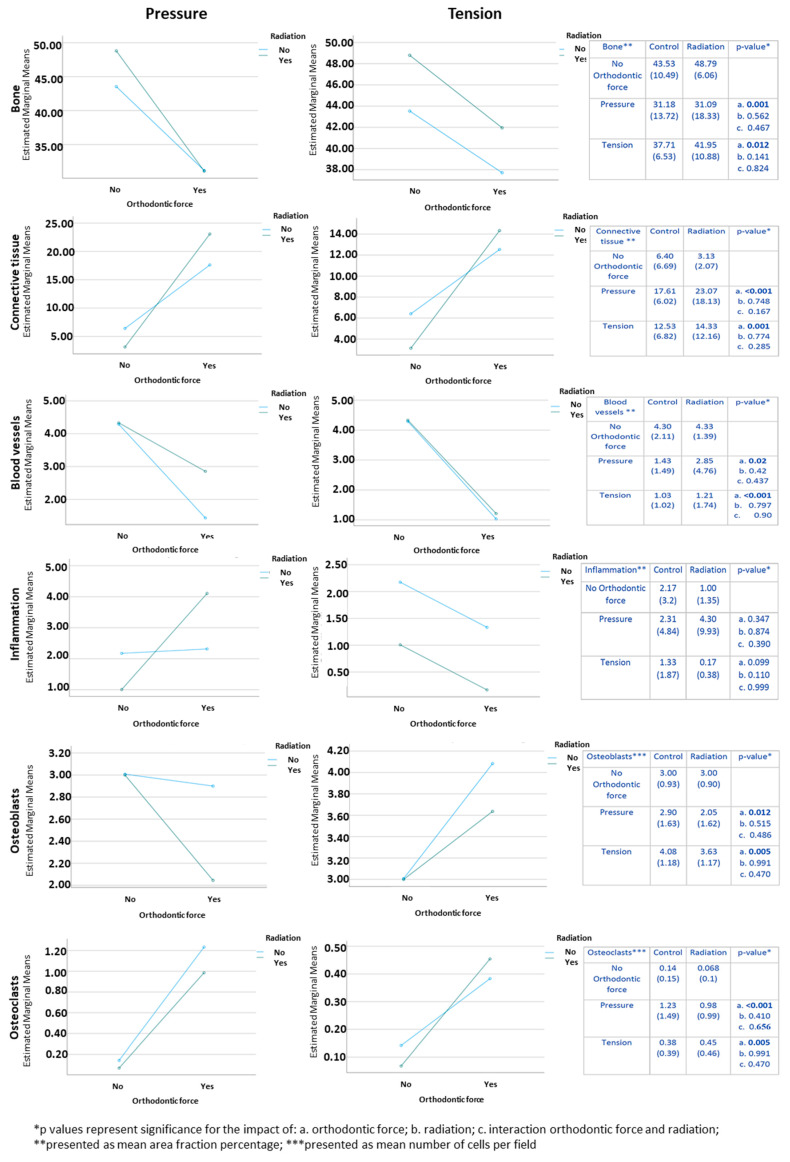
Histomorphometric changes in bone, connective tissue, blood vessels, inflammation, osteoclasts, and osteoblasts as a factor of orthodontic force and radiation.

**Figure 4:**
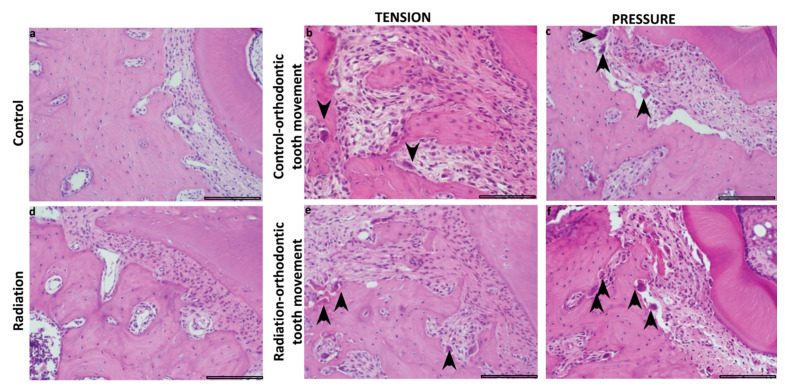
Representative sections showing multi-nucleated osteoclastic cells (arrows) as a factor of study group and tension/pressure sides (hematoxylin and eosin, scalebar 50 µ): (**a**–**c**) control group, (**d**–**f**) irradiated group. Note the increased number of osteoclastic cells in the pressure side in both groups.

In the pressure side, the mean area fraction of bone, connective tissue, and blood vessels and the mean number of osteoclasts were significantly affected by orthodontic force. The mean values of bone and blood vessels were lower in association with orthodontic tooth movement compared to those when no orthodontic force was applied, while the mean values of connective tissue and osteoclasts were higher in association with orthodontic tooth movement compared to those when no orthodontic force was applied (Figure 3). Inflammation and osteoblasts were not significantly influenced by orthodontic force. None of the parameters were significantly affected by radiation. Similar differences were found in the tension side, where we additionally found that the mean number of osteoblasts was higher in association with orthodontic tooth movement compared to the mean number when no orthodontic force was applied (Figure 3). Figure 4 highlights the osteoclasts in histopathological sections in the different study groups.

### 3.4. Immunohistochemical Stains

#### 3.4.1. Cells Expressing Markers of Pre-Osteoclasts: RANK, CD11b, and CD14

**Figure 5:**
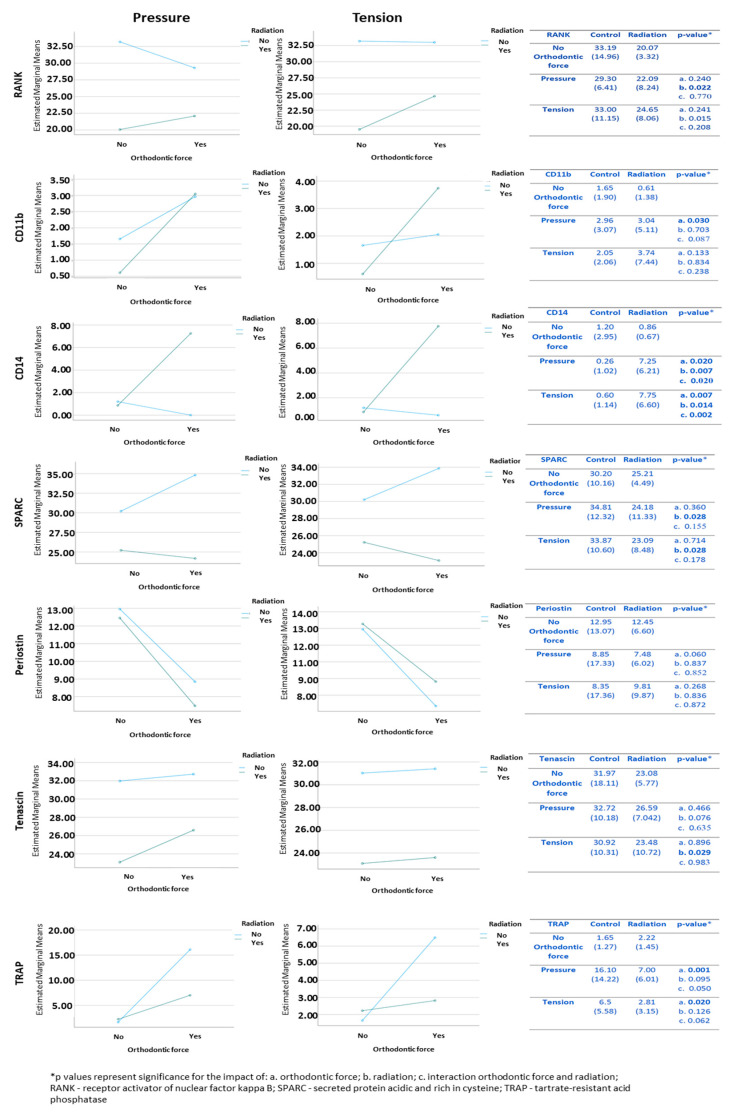
Changes in cells immuno-positive for pre-osteoclast markers (RANK, CD11b, CD14) and pre-osteoblast cells (SPARC, periostin, tenascin) as a factor of orthodontic force and radiation.

At the baseline (control; no orthodontic force), the frequency of these cells was differential, with RANK+ being the most common and CD11b+ and CD14+ being present in small amounts. In the pressure side, CD11b+ and CD14+ cells were significantly affected by orthodontic force, with the mean values being higher in association with orthodontic tooth movement compared to the mean values when no orthodontic force was applied. In addition, the CD14+ cells were significantly affected by radiation, with the mean values being higher in association with radiation compared to those when no radiation was applied (Figure 5). There was a significant interaction between orthodontic force and radiation in the CD14+ cells, with both factors being involved in the increase in the mean number of these cells in the radiated maxilla submitted to orthodontic force. RANK+ cells were also significantly affected by radiation, with mean values being lower in association with radiation compared to those when no radiation was applied. In the tension side, only the CD14+ cells were significantly affected by orthodontic force, with mean values being higher in association with orthodontic tooth movement compared to those when no orthodontic force was applied (Figure 5). In regard to radiation, it significantly influenced CD14+ cells, in a pattern similar to that of the pressure side, and a significant interaction was found between radiation and orthodontic force, where both factors were involved in the increase in the mean number of CD14+ cells in the radiated maxilla that underwent orthodontic force. RANK+ cells were also significantly affected by radiation, with mean values being lower in association with radiation compared with those when no radiation was applied.

#### 3.4.2. Cells Expressing Markers of Pre-Osteoblasts: SPARC, Periostin, and Tenascin

**Figure 6:**
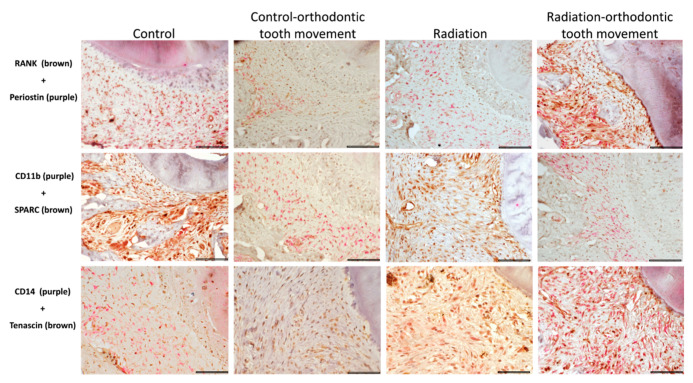
Pressure side: representative sections showing the various pairs of double immunohistochemical stains for identification of the expression of pre-osteoclastic and pre-osteoblastic markers in the various study groups (scalebar 50 μ).

**Figure 7:**
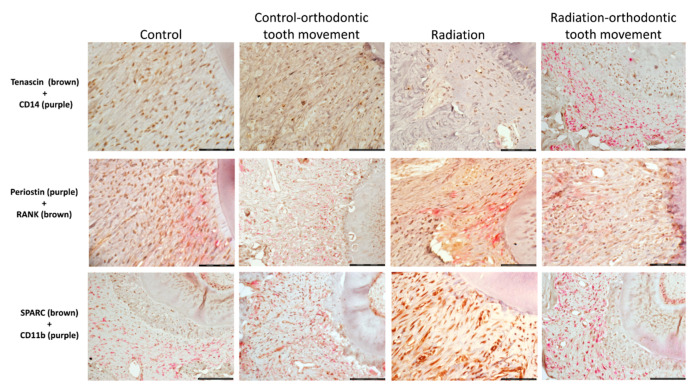
Tension side: representative sections showing the various pairs of double immunohistochemical stains for identification of the expression of pre-osteoclastic and pre-osteoblastic markers in the various study groups (scalebar 50 μ).

At the baseline (control; no orthodontic force), the frequency of these cells was differential, with SPARC+ being the most common and periostin+ and tenascin+ being fewer. In the pressure side, only SPARC+ cells were significantly influenced by radiation, with the mean values being lower in association with radiation compared to those when no radiation was applied (Figure 5). In the tension side, both SPARC+ and tenascin+ cells were significantly influenced by radiation, where the mean values were lower in association with radiation compared to those when no radiation was applied (Figure 5). Representative immunohistochemically stained sections of the different types of pre-osteoclast and pre-osteoblast cells in the study groups in the pressure side are displayed in Figure 6 and in the tension side in Figure 7.

#### 3.4.3. Tartrate-Resistant Acid Phosphatase (TRAP) Staining for Activity of Osteoclasts

**Figure 8:**
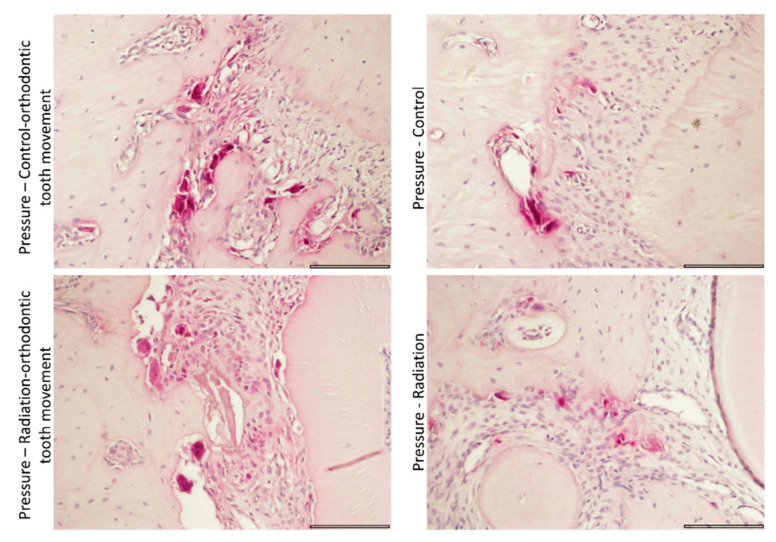
Representative sections of TRAP-stained sections for identification of active osteoclastic cells as a factor of study group in the pressure side (scalebar 50 μ).

TRAP+ cells were significantly affected by orthodontic force in both pressure and tension sides, with mean values being higher in association with orthodontic tooth movement compared to those when no orthodontic force was applied (Figure 5). Radiation had no significant effect. Figure 8 provides representative TRAP-stained sections in the study groups.

## 4. Discussion

The current study used an animal model designed to simulate orthodontic treatment in post-irradiated patients. The novelty of this work lies in its having investigated for the first time the impact of application of orthodontic force on irradiated maxillae in terms of changes in microarchitecture in parallel to corresponding changes in histomorphometry and phenotypes of pre-osteoclast and pre-osteoblast cells.

The micro-CT analysis (all parameters) showed that microstructural changes were significantly associated only with orthodontic force application and not with radiation. Nonetheless, the distance of orthodontic tooth movement in irradiated jaws was 30% less compared to that in non-irradiated jaws, assuming that changes induced by orthodontic force could restore a large part, but not all, of the irradiation-related changes. The partial recovery of the irradiated bone, as shown microstructurally, could be supported by the histomorphometric findings, especially regarding the increased number of osteoclasts, that was also associated with orthodontic force application. Using a molecular study, we were able to reveal for the first time the baseline differential distribution of cells expressing pre-osteoclast markers in the periodontal ligament, with RANK+ cells being the predominant population and CD11b+ and CD14+ cells being present in fewer numbers. Changes in these cells also seemed to occur in a selective manner, where orthodontic force was associated with an increase in CD11b+ and CD14+ cells and radiation was associated with a decrease in RANK+ cells. The activation status of osteoclasts (i.e., RANK+, CD11b+, and CD14+ cells) was assessed by TRAP staining, the increase in which was also related to the application of orthodontic force. The net balance between orthodontic force and radiation showed that although orthodontic force resulted in a significant increase in CD11b+ and CD14+ cells, it was the radiation-associated decrease in the predominant RANK+ cells that could explain the lack of complete bone recovery and impaired tooth movement in the irradiated rats.

There are only two publications, both of one group of researchers, with a study design partially similar to our current study [28,29]. However, key methodological differences can be noted between those studies and ours with regard to the brand of rats, irradiation source and dose and fractionation, orthodontic force, duration of orthodontic treatment, and selected tooth and area selected for investigation. In one study, the authors assumed that the rat periodontal ligaments are devoid of osteoclasts and that these cells would emerge rapidly from pre-osteoclasts that reside within the periodontal ligament once orthodontic force has been applied [29]. However, given that these progenitor cells are less differentiated and do not divide, they are less radiosensitive and are expected to undergo differentiation and maturation into activated osteoclasts once the orthodontic movement has been initiated. In addition, they suggested that osteoclasts would eventually be replenished from the bone marrow in case of prolonged orthodontic force. Others have claimed that in physiological conditions, once orthodontic force has been applied, the recruitment of pre-osteoclasts and their maturation occur within the bone marrow of the jaws, after which, they migrate to the periodontal ligament as multinucleated osteoclasts and that only their final activation occurs within this compartment [30,31]. In our histomorphometric assessment, at the baseline, we found a small number of multinucleated osteoclasts in the controls. However, the molecular study results showed that the periodontal ligament harbored abundant cells with the expression of pre-osteoclast cell markers. These cells showed a differential distribution, with the most frequent being RANK+ cells and CD14+ and CD11b+ being far less frequent. The total number of these cells, especially RANK+ and CD14+, changed little upon application of orthodontic force. In parallel, the TRAP-related activation state increased almost 10-fold (pressure side), suggesting that it may be the state of activation of mainly the RANK+ cells, rather than their numbers, that plays a critical role in orthodontic tooth movement. In irradiated rats, the baseline differential distributions of cells with pre-osteoclast markers were similar to those of the controls. However, in contrast to controls, the application of orthodontic force was related to a significant increase in CD14+ and CD11b+ cells, while the number of RANK+ decreased. Accordingly, we suggest that orthodontic tooth movement in the controls was based on the maturation and activation of resident RANK+ cells within the periodontal ligament, without major recruitment of pre-osteoclasts from other sources. Yet, when orthodontic force was applied in irradiated rats, CD14+ and CD11b+ cells were recruited from sources outside the periodontal ligament. Given the radiation-associated decrease in RANK+ cells, which are the predominant pre-osteoclast cells, it would appear that the net balance tended to be toward impaired bone absorption. These findings can serve as a feasible explanation for why the distance of orthodontic tooth movement in the irradiated rats was 30% less than in the controls.

There is only one study that has investigated the presence of CD11b+ cells in the periodontal ligament of rats in experimental orthodontic tooth movement [32]. In this study, CD11b+ cells were assumed to be of myeloid-born macrophages/dendritic cell lineage. It was found that normal rat periodontal ligament hosted high numbers of macrophage- and dendritic-like CD11b+ cells and only a few lymphocytes and granulocytes and that experimental tooth movement resulted in significant recruitment of cells belonging to the mononuclear phagocytic system but had no significant effect on the number of lymphocytes and granulocytes. These findings are not entirely in agreement with our current results, since we found that the number of CD11b+ cells in the controls was low but that it increased upon application of orthodontic force.

Cells expressing pre-osteoblast markers (SPARC and tenascin) were negatively affected by radiation but not by orthodontic force. In general, there is considerable controversy regarding the response of osteoblasts to irradiation, and their mechanisms of response are still poorly understood [33]. In vitro studies have shown a wide range of effects of radiation on their viability, proliferation, and differentiation [34]. Some studies have found that osteoblasts remain viable after irradiation with doses between 10 and 30 Gy [35,36], while others have argued that osteoblastic viability is already impaired at doses of 4 [34] and 5 Gy [33]. Nevertheless, in spite of inhibition of osteoblastic proliferation, it has been found that irradiation in a range of 4 to 8 Gy enhances the expression of genes associated with the production of proteins regarded as osteoblastic markers, including collagen I, alkaline phosphatase, Runx2, osterix, and osteocalcin [33,37]. In our study, we also found a negative influence of radiation on pre-osteoblast-associated cells, which was not compensated by orthodontic force. This seems to be in line with the negative effect that we found on the mean area fraction percent of bone even in the tension side, where bone formation would have been expected.

In our study, periostin, as a marker of pre-osteoblasts, was present in the periodontal ligament of our control rats, in both tension and pressure sides. Its expression was not significantly affected by either orthodontic force or irradiation. Other studies that have investigated the expression of periostin in the setting of orthodontic tooth movement but not in the context of irradiation have reported similar findings in both in vitro and in vivo settings [38,39,40,41,42]. It seems that periostin is one of the local contributing factors in bone and periodontal tissue remodeling following mechanical stress during experimental tooth movement. The importance of its activity was assessed in periostin-null mice in which the distance of the tooth movement and mineral deposition rates were significantly reduced and the arrangement, digestion, and integrity of collagen fibrils were impaired [40,41].

Tenascin and orthodontic tooth movement have been investigated in only a few studies. Its expression was found to be increased in both tension and pressure regions of the periodontal ligament under orthodontic load compared with unloaded controls [43,44]. We found the expression of tenascin to be negatively affected by radiation, but this initial finding is awaiting further investigation.

SPARC, as a marker of pre-osteoblasts, was presently found to be abundantly expressed in the periodontal ligament of our control animals. Radiation had a negative effect on cells expressing SPARC. The expression of SPARC in orthodontic tooth movement has been scarcely investigated. Interestingly, in one in vitro study, it was found that the SPARC gene was among several force-sensitive genes related to the extracellular matrix and adhesion in stretched human periodontal ligament cells, probably playing a role in the stretch-induced cell realignment and mechanic force related to periodontal remodeling [45]. The precise role of SPARC in orthodontic tooth movement, however, also awaits further investigation.

The strength of the present study lies in its use of different methodologies and multiple molecular markers for cells with a phenotype of pre-osteoclasts and pre-osteoblasts for investigating the changes induced by radiation and orthodontic tooth movement. The limitations of this study are that a single, relatively low dose of radiation was used and that the assessments were performed at one time point. The current study did not consider the additional effects of radiation, such as oral mucositis, salivary flow rate, changes in the oral microflora, and dysgeusia, all of which can be encountered in the clinical setting.

## 5. Conclusions

Our study has shown for the first time that the periodontal ligament harbors different populations of pre-osteoclast cells and that orthodontic force and radiation seem to exert different effects on them. The overall balance showed that orthodontic force elicits a substantial microstructural, histological, and functional normalization process in irradiated jawbones but that radiation-induced damage is still conspicuous. Further studies are needed to validate our findings and to add to our understanding of the orthodontic force-radiation inter-relations.

## Figures and Tables

**Table 1:** Antibodies used for the immunomorphometric assessment of pre-osteoblast/osteoblast and pre-osteoclast/osteoclast cell phenotypes.

	Coupled Antibodies		Coupled Antibodies		Coupled Antibodies	
	RANK ^1^	Periostin	CD14	Tenascin C	CD11b	SPARC ^2^
**Source of antigen; manufacturer; concentration**	Mouse; Novus Biologicals, Centennial, CO, USA; Catalog number NB100-56508; 1:200	Rabbit; Novus Biologicals, Centennial, CO, USA; Catalog number NBP1-30042; 1:200	Rabbit; Bioss, Woburn, MA, USA; Catalog number bs-1192R; 1:250	Mouse; Novus Biologicals, Centennial, CO, USA; Catalog number NB110-68136H; 1:40	Rabbit; OriGene, Rockville, MD, USA; Catalog number TA323950; 1:50	Rabbit, Proteintech Group, Rosemont, IL, USA; Catalog number 15274-1-AP; 1:100
**Identified phenotype: pre-osteoblasts/osteoblasts**		+		+		+
**Identified phenotype: pre-osteoclasts/osteoclasts**	+		+		+	
**Cellular location of target antigen**	Cytoplasm	Mainly extracellular matrix (as secreted protein) tissue); also cytoplasmic [20,21]	Cell surface	Mainly extracellular matrix (as secreted protein); also cytoplasmic [21,22,23]	Cell surface	Cytoplasmic and extracellular matrix (as secreted protein) tissue) [21,24,25,26]

^1^ RANK: receptor activator of nuclear factor kappa B; ^2^ SPARC: secreted protein acidic and rich in cysteine.

## Data Availability

The data presented in this study are available on request from the corresponding author.
